# Integrative Alternative Tactics for Ixodid Control

**DOI:** 10.3390/insects13030302

**Published:** 2022-03-18

**Authors:** Allan T. Showler, Perot Saelao

**Affiliations:** Knipling-Bushland U.S. Livestock Insects Research Laboratory, USDA-ARS, Kerrville, TX 78028, USA; perot.saelao@usda.gov

**Keywords:** acaricides, botanical, cultural control, desiccant dust, insect growth regulator

## Abstract

**Simple Summary:**

Hard ticks are important for economic and health reasons, and control has mainly relied upon use of synthetic acaricides. Contemporary development of resistance and concerns relating to health and environmental safety have elicited exploration into alternative tactics for hard tick management. Some examples of alternative tactics involve biological control, desiccant dusts, growth regulators, vaccines, cultural methods, and ingested medications.

**Abstract:**

Ixodids (hard ticks), ectoparasitic arthropods that vector the causal agents of many serious diseases of humans, domestic animals, and wildlife, have become increasingly difficult to control because of the development of resistance against commonly applied synthetic chemical-based acaricides. Resistance has prompted searches for alternative, nonconventional control tactics that can be used as part of integrated ixodid management strategies and for mitigating resistance to conventional acaricides. The quest for alternative control tactics has involved research on various techniques, each influenced by many factors, that have achieved different degrees of success. Alternative approaches include cultural practices, ingested and injected medications, biological control, animal- and plant-based substances, growth regulators, and inert desiccant dusts. Research on biological control of ixodids has mainly focused on predators, parasitoid wasps, infective nematodes, and pathogenic bacteria and fungi. Studies on animal-based substances have been relatively limited, but research on botanicals has been extensive, including whole plant, extract, and essential oil effects on ixodid mortality, behavior, and reproduction. The inert dusts kaolin, silica gel, perlite, and diatomaceous earth are lethal to ixodids, and they are impervious to environmental degradation, unlike chemical-based toxins, remaining effective until physically removed.

## 1. Introduction

Ixodids are important vectors for a wide range of disease-causing agents that infect humans, pets, livestock, and wildlife [1] and the pests are also associated with reduced meat and milk production, declines in reproductive capacity, damage to hides, and host mortality; all of which result in substantial financial losses for the livestock, particularly cattle, industry.

As ectoparasites that feed on the blood of hosts, ixodids can facilitate rapid disease transmission from infected hosts to healthy ones, exposing the healthy animals to a wider variety of pathogens than any other group of arthropods [2,3]. Abundances of ixodids, their population dynamics, and land management practices can directly influence the transmission of tick-borne pathogens [4].

As an example, bovine babesiosis is a tick-borne parasitic disease that causes considerable financial losses to the cattle industry. *Babesia bovis* (Babés, Piroplasmida: Babesidae) and *B. bigemina* Smith and Kilborne [5] are protozoan hemoparasites that cause most clinical cases of bovine babesiosis [6]. Babesiosis results in substantial morbidity and mortality in cattle with an estimated financial impact of >USD 17 million in Asia, Africa, and Australia [7].

Anaplasmosis is a vector-borne disease resulting from an infection by the bacterium *Anaplasma phagocytophilum* (Rickettsiales: Anaplasmataceae) and *A. marginale* [8]. *Anaplasma phagocytophilum* Telford (Rickettsiales: Anaplasmataceae) is the primary bacterium responsible for infections of humans [9], while *Anaplasma marginale* is typically responsible for infections of cattle. All of these *Anaplasma* species are vectored by ixodid spp. [10].

Ixodids are also responsible for transmission of human diseases. Ixodid-borne transmission of pathogenic agents presents public health concerns on a global scale [11]. *Ixodes scapularis*, *I. pacificus*, and *I. ricinus* are the primary vectors of *Borrelia burgdorferi* sensu lato and *A. phagocytophilum*, the causative agents of Lyme disease and human granulocytic anaplasmosis, respectively [12]. Tularemia is another ixodid-borne disease caused by the bacterium *Fancisella tularensis* [13]. The type A strain is solely found in North America with a mortality rate of up to 60% when left untreated [14]. Recently, nearly half of all United States tularemia cases were ixodid-associated [3]. As an additional example, typhus is another ixodid transmitted disease caused by *Rickettsia prowazekii*. This pathogen makes its way into the gut epithelium of the ixodid, passing into the feces where it can then infiltrate humans through skin abrasions by contact with infected feces [15].

Application of conventional synthetic acaricides has been the main tactic for controlling ixodid infestations [16,17]. A method from the 1950s to the 1980s involved area-wide broadcast applications using sprayers and dispersion of toxicant-augmented granule formulations for reducing *A. americanum* and *I. scapularis* infestations [16,17,18,19]. Those formulations, often highly volatile, varied in terms of frequency of usage, seasonality, and efficacy (from 64% to 100%) [20,21]. In addition, many of the synthetic acaricide formulations were organophosphate-based, which are no longer in use in residential areas due to their toxicity to nontarget organisms, including humans [22]. Most contemporary acaricides involve pyrethroid and carbamate compounds as active ingredients [22], which can confer substantial benefits (e.g., relatively rapid mortality and strong efficacy) when used effectively [22]. In studies on acaricide treatment of American dog ticks, *Dermacentor variabilis* (Say), control rates have shown 82%, 95%, and 96% reductions after one, two, and three treatments, respectively [23]. Conventional synthetic acaricides are efficacious against multiple ixodid species [20,21], but over-reliance on synthetic acaricides risks the development of ixodid resistance; a two-year study found that nearly 60% of *Rhipicephalus appendiculatus* and *Rhipicephalus decoloratus* treated with cypermethrin and deltamethrin (both pyrethroids) survived [24]. As an alternative to spray treatments, dipping vats are a common treatment option for many producers to apply the synthetic chemical coumaphos [25]. Treatments using coumaphos through dipping have shown effective control rates of >99% when not followed by rainfall [25]. However, ixodid resistance to synthetic acaricides, such as coumaphos, has been reported as early as during the 1990s [26]. In one study [26], for example, despite increasing the coumaphos dose nearly threefold, no difference in larval ixodid mortality was observed. Miller et al. [27] suggested that organophosphate resistance in *R. microplus* required high-sode treatments to overcome resistance. Research into naturally derived compounds such as desiccant dusts [28,29,30,31,32] have been showing promise as alternatives to overcome the weaknesses inherent to synthetic acaricides.

While conventional synthetic acaricides have been the mainstay of ixodid control, the possibility of resistance to those acaricides, environmental and human health concerns, and costs are some issues that suggest that alternative tactics might enhance control, particularly if those tactics are integrative with conventional synthetic acaricides and with one another. The purpose of this review, using selected examples to represent a greater whole, is to describe alternative integrative tactics for ixodid control.

## 2. Growth Regulators

In response to increased resistance of ixodids to conventional acaricides [33,34,35], alternative chemically-based acaricides are being sought [35,36,37,38,39]. Fluazuron was the first growth regulator registered for ixodid control (Acatak, Novartis, Basel, Switzerland) [40,41], with an LD_50_ and LD_95_ of 19.5 mg/kg and 100 mg/kg, respectively, against engorged *R. sanguineus* nymphs when used as a pour-on to rabbits [41]. Novaluron is a benzoyl urea with low toxicity to most nontarget organisms, including mammals, birds, and earthworms [42,43,44]. It inhibits chitin synthesis on many insects [42,43,44,45,46,47,48,49,50,51], and it was tested, with inconsistent results, on mites [52,53,54,55] and *R. microplus* [56]. The differences in observed efficacy suggest that additional testing is likely desirable.

Pyriproxyfen is a juvenile hormone analog used for flea control [57,58,59]. It had variable results when applied to mites [60,61,62] et al. and ixodids [63,64,65].

Tekko Pro, sold as an insect growth regulator, contains 1.3% novaluron and 1.3% pyriproxyfen [66], but is not registered for use against acarines. Engorged larval *R. sanguineus* molting in a nontreated control was 1.2-, 2.1-, and 2.9-fold greater than on substrate treated with 4 µg/cm^2^, 8 µg/cm^2^, and 16 µg/cm^2^ (total a.i.) Tekko Pro [67]. At the high concentration, ≈35% of the larvae molted while molting of engorged nymphs was not reduced, but eggs from treated females failed to hatch, and all of the females died before most of their eggs were deposited [67]. Tekko Pro was more potent against *A. americanum* than *R. sanguineus*; molting larval and nymphal *A. americanum* decreased >95% and up to 90%, respectively [67]. On treated calves, 99% of *R. microplus* larvae did not develop into adults [67]. The product protected calves for at least 30 d (and <52 d) [67]. Growth regulators likely offer an effective, nonconventional means of ixodid control on host animals.

## 3. Botanically-Based Substances

Extracts and oils produced by plants have been extensively researched for acaricidal properties. Bioactive substances occur in leaves, stems, bark, roots, flowers, and seeds. Effects of extracts are influenced by ixodid species, life stage, exposure time, solvent, method of extraction, concentration, presence of additional bioactive components, ixodid feeding status, in vitro vs. in vivo, and other factors. Modes of action vary as well, and include interference with nerve transmission, respiration, and cell membrane permeability.

Studies assessing botanically-based substances have likely been conducted on all economically and medically important ixodid species; we present representative examples suggesting the diversity of research on, and effects of, botanical substances applied for ixodid control (Table 1 and Table 2). Broadly, there are three categories of botanically-based substances: plants in situ, extracts, and essential oils. Effects of botanically-based substances induce mortality and sublethal effects, such as repellency, deterrence, growth regulation, and impaired reproductive potential.

Plant-based substances have desirable qualities that include relatively fast action, certified organic status for consumers of organic agricultural products, and extracts and essential oils can have multiple bioactive components with different modes of action, making the development of resistance to the substance less likely than to a single bioactive compound. Disadvantages of botanical substances can, in some instances, involve high cost of production and the need to culture the plant species on a sufficiently large scale, relatively short residual efficacy, possible nontarget effects, and variability in terms of efficacy due to plant genotypic differences, soil type, quality and quantity of fertilizer, and other factors that affect plant physiochemistry and health.

### 3.1. Plants In Situ

Certain intact and growing grasses have anti-ixodid properties. Japanese stiltgrass, *Microstegium vimineum* (Trin.) A. Camus, increased mortality of *A. americanum* and *D. variabilis* by 173% and 70%, respectively [198]. Aycardi et al. [199] found that female ixodids in pastures and on pastured cattle were more abundant in association with signal grass, *Brachiaria decumbens* Stapf, than on gamba grass, *Andropogon gayanus* Kunth, and particularly molasses grass, *Melinus minutiflora* P. Beauv. For reducing ixodid populations, Thompson et al. [200] suggested that *M. minutiflora* and *A. gayanus* should be planted in infested areas. Fernandez-Ruvalcaba et al. [201], using *A. gayanus* and *M. minutiflora* plots, reported that questing *R. microplus* larvae were 52.1% and 73.3% less abundant than on nonrepellent and nonacaricdial grass species (as determined by flag sampling [28]). All *R. appendiculatus* instars avoid climbing on *M. minutiflora* but not on kikuyu grass, *Pennisetum clandestinum* Hochst. ex Chiov [118]. Although *M. minutiflora* reduces ixodid survival, the effect is relatively minor and slow [202], and plots of *M. minutiflora* experimentally infested with larval *R. microplus* were repellent only when the plants were ≥6 months old [201] (4). While in situ plant growth for ixodid control appears promising and has not been associated with ill effects on cattle, it has not been adopted commercially.

### 3.2. Extracts

Extracts are obtained by a variety of methods involving solvents that include water, ethanol, methanol, hexane, chloroform, and carbon dioxide, each of which can result in different constituents and concentrations of bioactive substances. The best known and most widely applied botanical extracts are pyrethrins, constituents of pyrethrum (extracted from the pyrethrum daisy, *Chrysanthemum cinerariaefolium* (Trevir.) Vis.) that account for its insecticidal activity [203,204,205]. Pyrethrins stimulate nerve cells by interfering with the voltage-dependent nerve membrane sodium channel which causes uncontrolled repeated discharges, culminating in paralysis and mortality [204]. Synthetically manufactured pyrethrin analogs, pyrethroids, the active ingredients of many commercial pesticides, affect nerve impulse transmission in the same way as pyrethrins [206]. Contemporary mainstays for controlling numerous pests of livestock, pets, and humans [204], pyrethroids have been increasingly weakened by the development of ixodid resistance against pyrethrins and pyrethroids, particularly apparent in *Rhipicephalus microplus* and *R. bursa* [37,207,208,209,210,211].

Pyrethrins, however, were found to be 100% effective against *A. americanum*, *A. cajenennense*, and *A. maculatum* within minutes [95]. The addition of pyrethrins to a silica gel-based desiccant dust (a commercial product, Drione) killed *A. americanum* larvae and nymphs substantially faster than the silica gel component alone [31], and, unlike silica gel, the product also killed *A. americanum* larvae and nymphs while they fed on cattle [29].

Extracts from the neem tree, *Azadirachta indica* A. Juss, have been widely investigated for pest management applications, including ixodid control. Immersion of adult female *R. microplus* for 5 min in 2% alcoholic *A. indica* extract (wt/vol) induced as much as 65% mortality [76]. Adult *R. microplus* were immersed for 1 min in *A. indica* ethanol extracts from leaves, bark, and seed; the 7% seed extract caused 80% mortality by 5 h, leaf extracts produced 30% kill, and bark extracts had no effect [88]. Weekly spot treatments using of 10% aqueous neem seed extract reduced *A. hebraeum*; small smooth bont-legged ticks, *Hyalomma truncatum* Koch, and *R. evertsi* on goats by up to 86.4%, 87.8%, and 84.2%, respectively [82]. Neem extracts contain azadirachtin and often other bioactive substances [80]; Mulla and Su [212] reported that neem extracts can have >35 different bioactive compounds that, in combination with azadirachtin, exert sublethal effects against ixodids, such as antifeedancy, deterrence [80], growth regulation, reduced fecundity, sterilization [83,87,88], oviposition repellency or attractancy, and changes in biological fitness [81,83,84,88,118,145].

Instead of describing each individual plant species and the effects of their extracts, we have listed selected studies that are illustrative of effects on ixodids (Table 1). The list includes 84 species of plants in 74 genera, and 16 ixodid species in six genera.

### 3.3. Essential Oils

Essential oils of plants are obtained by processes that include water distillation, steam distillation, cohobation, maceration, and enfleurage. Similar to many other botanical substances, they can be useful for cooking, aroma production, medicine, and pest management.

While numerous plant species produce essential oils with acaricidal properties (Table 2), lemon grass, *Cymbopogon citratus* Stapf, is arguably the most well-known. The essential oil can contain multiple bioactive compounds, such as citral α, citral β, netol geraniol, citronellal, terpinolene, geranyl acetate, myrecene, and terpinol methylheptenone [213]. *Cymbopogon citratus* essential oil inhibited *R. microplus* oviposition and egg hatch by up to 66% and 100%, respectively [175], and, in a different study, the oil inhibited *R. microplus* oviposition, egg hatchability, and egg weight [214]. Essential oil of *C. citratus* also caused 98%, 100%, and 96% mortality of Asian longhorned tick, *Haemophysalis longicornis* Neumann, adults, nymphs, and larvae, respectively [117].

Essential oil of *A. indica* has also received attention for acaricidal effects. Neem seed oil induced complete mortality against larval *A. variegatum* by 48 h, and the effects were dose- and time-dependent [157]. Abdel-Shafy and Zayed [158] reported that although neem seed oil increased the hatching rate of *H. anatolicum excavatum* eggs during the first 7 d after application, the larvae were incompletely developed and dead, and hatching ceased by 15 d. Moreover, mortality of nonfed larvae and nonfed adults was 100%, and 1.6% to 3.2% concentrations were suggested for commercial ixodid control [158]. Mortality of engorged adult female *R. microplus* reached 82.6% 15 d after application of 12% *A. indica* oil, egg hatch decreased by 59.4% [160] and, while *A. indica* had no effect on *H. dromedarii* egg production and adult feeding, larval molting declined by 60% [159].

Essential oils of cedar species have also received substantial research attention for acaricidal effects. Incense cedar, *Calocedrus decurrens* (Torr.) Florin; Port Orford cedar, *Chamaecyparis lawsoniana* (A. Murr.) Parl.; and western juniper, *Juniperus occidentalis* (Hook), oils were biocidal to *I. scapularis* nymphs [163]. Essential oil Alaska yellow cedar, *Chaemaecyparis nootkatensis* (D. Don) Spach, heartwood, containing the bioactive monoterpene carvacrol, had an LC_50_ of 0.00068% 24 h after application to *I. scapularis* nymphs, and nootkatone, an eremophilane desquiterpene, had an LC_50_ of 0.0029% [168]. Dietrich et al. [169] demonstrated that nootkatone and valencene-13-ol from *C. nootkatensis* oil had repellency RC_50_ values of 0.086% and 0.112%, respectively, against *I. scapularis* nymphs, compared to DEET’s RC_50_ of 0.073%.

## 4. Animal-Based Substances

There has only been one study on ixodid control using natural animal products. Formic acid, produced in ants of subfamily Formicinae, is a volatile one-carbon molecule used for trail marking and defense. It acts as an airborne toxin and signals alarm [215,216,217]. Rolling on ants, or “anting”, by some birds and mammals [218,219,220,221,222,223], is claimed to work formic acid onto hair and feathers to protect against ectoparasites, although evidence is lacking in terms of ixodids [216]. Formic acid occurs naturally in honey, which can become sufficiently concentrated to achieve ≥95% control of the varroa mite, *Varroa destructor* Anderson & Trueman [224], a serious pest of honeybee, *Apis mellifera* L., colonies [225,226,227]. Used as a fumigant in *A. mellifera* colonies, formic acid binds cytochrome c oxidase in mitochondria, obstructing energy metabolism [228] and instigating neuroexcitation [229]. Registered with Section 3 [230] approval in the United States, the compound is the active ingredient of commercial products for *V*. *destructor* management [231].

Immersion of *A. americanum* larvae and nymphs for ≈10 min in a 5% concentration of formic acid in acetone solvent killed ≈5.9-fold more larvae and nymphs than the acetone control [232]. Contact by crawling on dried 1% formic acid-treated filter paper for 10 min under ventilated conditions killed 50–100% of *A. americanum* larvae by 1 h and mortality was 83.6–99% by 4 h; when larvae were exposed to the treated substrate for 30 min and then removed, mortality was 94–100% by 1 h [232]. Used as a fumigant in enclosed containers, 1% formic acid achieved complete larval kill, and nymphal mortality, by 20 min, was ≥8.1-fold greater than in the control [232]. Formic acid’s efficacy as a fumigant is due to action on the respiratory system but treating ixodids with lethal doses of volatiles where ixodids naturally occur does not appear to be practical [232]. As a contact acaricide, formic acid might be more lethal when combined with a lipophilic adjuvant to enhance penetration of the ixodid cuticle [232]. Although formic acid was claimed to repel *Amblyomma incisum* Neuman nymphs and *Amblyomma parvum* Aragão adults [216], and Zingg et al. [217] reported that questing ixodid populations were negatively correlated with nearby red wood ant, *Formica polyctena* Först., colonies, Showler et al. [232] did not observe repellency or deterrence in laboratory bioassays.

## 5. Injected and Ingested Curative Medications

Ingestible and injectable medications offer an alternative ixodid treatment option. A compound known as fluralaner, an isoxazoline acaricide that is systemically distributed post-ingestion and highly selective in its activity against ectoparasites, is widely used for controlling ixodids on dogs and cats, applied orally and topically. Isoxaloline blocks and inhibits arthropod γ-aminobenzoic acid (GABA) and glutamate-ligand gated chloride channels [233,234,235], acting as a noncompetitive GABA-receptor antagonist and toxicant to arthropod neurons [236]. Fluralaner can be rapidly absorbed through the host’s intestinal tract shortly after oral administration and attains maximum plasma concentrations within 24 h, remaining detectable for up to 116 d post-treatment [236].

Other compounds are more widely used in the cattle industry, lowering ixodid fecundity and reducing numbers of ixodid infested cattle. Closantel, N-[5-chloro-4-[(4-chlorophenyl)cyanomethyl]-2-methylphenyl]-2-hydroxy-3, 5-diiodobenzamide, is a halogenated salicylanilide that has strong antiparasitic activity [237], particularly on cattle when applied by subcutaneous injection and ingestion [238]. Closantel can provide >90% reduction of *A. americanum* [238].

Macrocyclic lactones are effective for controlling ixodids on many animal hosts, applied through ingestion and injection [239]. Several forms of macrocyclic lactones exist, but the two most commonly used are doramectin and ivermectin [240,241]. Ivermectin is efficacious against ectoparasitic fleas, flies, ticks, and mites [242]. Subcutaneously injected ivermectin reduced numbers of *R. microplus*; face flies, *Musca autumnalis* DeGeer; horn flies, *Haematobia irritans* (L.), and *Orthellia cornicina* (F.) on cattle [25,243]. Ivermectin applied as treated corn fed to white-tailed deer reduced on-host *A. americanum* adults and nymphs by 83–92%, and clusters of larvae on vegetation were eliminated [37,244,245]. Further, single ivermectin injections reduced female *R. microplus* by 90–95%, and numbers of females that survived to repletion to by 99% [25]. Doramectin, a macrocyclic lactone often derived through fermentation from *Streptomyces avermitilis* Omura, is another medication that has shown a post-treatment ixodid control rate of 99% at 4 d post treatment on cattle [246]. Doramectin reduced the total number of engorged female *R. microplus* by 51% at 24 h post-treatment, with an increase in efficacy of up to 99% by 4 d [246]. Other studies have found efficacy rates of 90–99% [247].

## 6. Vaccines

Vaccines that target ixodids have shown limited potential as an alternative approach for managing ixodid infestations [248,249], offering a tactic that might avoid environmental risks associated with conventional synthetic toxin-based pesticides. Additionally, vaccines can complement other ixodid control tactics with the aim of mitigating the development of resistance to acaricides [250] Vaccines can be designed to deploy multiple antigens, enabling protection for a wide range of host species against multiple ixodid species [251]. As an example of current efforts to develop anti-ixodid vaccines, candidate vaccines are being investigated with the goal of providing prophylactic protection of cattle from *R. microplus* [252], while recent efforts using recombinant antitick vaccines afforded nearly 82% efficacy against tick infestation [253]. Additional vaccine development opportunities exist, with a current focus on improving identification and characterization of ixodid antigens that can then be generated in the laboratory [254]. Further, relatively recent research has identified compounds within ixodid saliva that might play functional roles in vaccine development [255]. Ixodid saliva warrants scrutiny because it contains proteins that facilitate transmission of ixodid-borne pathogens through the salivary protein’s anti-inflammatory, anticoagulant, and immunosuppressive properties [255,256]. Several ixodid salivary gland proteins (TSLPI, Salp15, tHRF, and TIX-5) have, as well, been identified as novel candidates for an anti-ixodid vaccine [257].

Most current research on candidate vaccines for protection against *B. burgdorferi* focuses on recombinant proteins, while DNA-based vaccines offer another vaccination research avenue [258]. DNA vaccines are relatively simple to produce, highly stable, and they might induce both humoral and cellular immune responses [259]. One candidate DNA-based vaccine, for instance, showed potential for protecting against *B. burgdorferi* by targeting the ixodid vector 249,257].

Vaccines targeting specific ixodid antigens appear to be a promising, safe, and environmentally friendly method in the laboratory [247,260]. However, few successes have been reported to occur under field conditions. Currently, only two Bm86-based vaccines have provided effective protection in the field and have been commercialized [261,262,263]. The potential of vaccines continues to develop along with improvements in the computational and with genomic methods used to identify target antigens.

## 7. Biological Control

### 7.1. Natural Enemies

A substantial range of generalist predators, that includes arachnids, crustaceans, insects, reptiles, amphibians, birds, and mammals, attack various ixodid life stages [264,265,266,267,268,269]. While it is commonly assumed that natural enemies have a robust governing effect on many arthropod populations, efficient natural enemies of ixodids, for the most part, have not been reported. As an example, pitfall traps deployed in a variety of habitats on the South Texas coastal plain during fall, winter, spring, and summer captured negligible quantities of carabid beetles and lycosid spiders, and sweep net samples of vegetation in the same habitats yielded a few linyphiid and salticid spiders, and a single reduviid [269].

Ants are, generally, efficacious natural enemies of arthropod pests [270,271,272,273,274]. Although ants, particularly the red imported fire ant, *Solenopsis invicta* Buren, have been associated with reduced ixodid populations [267,270,275,276,277,278,279,280], evidence for direct effects on ixodids has largely been circumstantial. While some ixodid species might be attacked by ants, metastriate ixodids (identified by a flap covering the sexual orifice) in the genera *Amblyomma*, *Dermacentor*, and *Rhipicephalus* (it is unclear as to whether cattle fever ticks, *Rhipicephalus (Boophilus)* spp., are included) [281], have dermal wax glands that secrete protective allomones [282,283,284]. The allomones occur in larval, nymphal, and adult metastriate ixodids; blood-engorged adults, however, might be more vulnerable to ants than nonfed adults and other life stages [282,283,284]. Barré et al. [285], however, observed that 8% of engorged tropical bont ticks, *Amblyomma variegatum* (F.), were attacked by tropical fire ants, *Solenopsis geminata* (F.). Another study found that blood-engorged adult lone star ticks, *Amblyomma americanum* (L.), were not recognized as prey by eight species of ants, including *S. invicta*, in three regions of Texas [286]. Red harvester ants, *Pogonomyrmex barbatus* (Smith), removed blood-engorged adults from the cleared area around the nest entrance, discarding them outside the clearing akin to foreign items, such as plastic vial caps [286]. Metastriate ixodid allomones appear to mask individuals from ants; hence, they are not recognized as prey [283]. The allomonal secretion (contains squalene but the active substances have not been verified) is purportedly elicited by pressure applied to the ixodid’s body, such as an ant’s bite on a leg; the secretory cells then “reload” for ≈10 d to full capacity [283]. Wax glands around the pressure respond by discharging allomone droplets while the other wax glands on the ixodid’s body remain “loaded” [283]. When all of the wax glands are discharged, and when adults are engorged with host blood, ixodids were reported to become vulnerable to ants [283]. Yoder et al. [283] suggested that swelling of the metastriate ixodid’s body creates sufficient integumentary pressure to discharge the wax glands, but predatory ants failed to recognize blood-engorged, intact adult *A. americanum* as prey [286]. The relative slow movement of engorged females, depletion of the allomones in metastriate ixodid wax glands due to abdominal expansion, and the possibility that ingested blood increases attractiveness to ants, might weaken masking [283]. In natural habitats, *A. americanum* larvae, nymphs, and nonfed and engorged adults, without physical pressure applied, were not attacked by ants. Injured engorged adult *A. americanum*, however, were attacked by predatory ants at the site of open wounds [286].

The metastriate ixodid female’s organ of Géné coats the eggs with a waxy antidesiccant during oviposition [287]. Indifference of ants, including *S. invicta*, to *A. americanum* eggs suggests that the waxy coating contains a masking allomone, possibly the same substance that is secreted from the integumentary wax glands [269,286]. When *A. americanum* eggs were crushed and presented to predatory ants, the ants also failed to recognize the mashed eggs as food [286].

Regardless of the wide distributions of predatory formicid species [288,289], ixodid populations persist where ants are abundant [290]. As an example, predatory ant species of the South Texas coastal plain do not eliminate ixodids despite the ubiquity and intensity of ant foraging recorded on meat- (hot dog) and insect-based (dead house flies, *Musca domestica* L.) baits [286,291]. Metastriate ixodids have adapted to defend against predatory ants, explaining, at least in part, how many ixodids can exist in relatively high populations alongside substantial populations of predatory ants [290].

While it is possible that metastriate ixodids might be vulnerable to attack from predatory ants when alternative food items are scarce, ants in South Texas did not recognize any life stage of *A. americanum* even during winter [286] when other prey was unlikely to be as profuse as during warmer seasons. In that region, temperatures are mostly >24 °C, excluding short, isolated periods when cold fronts pass through. Prey availability in that environment is relatively great; hence, it is not likely that predatory ants will be compelled to feed on metastriate ixodids.

A laboratory study showed that the Australian *Rhytidoponera* sp. ant preyed upon two ixodids, *Aponomma hydrosauri* (Denny) and *Amblyomma limbatum* Neumann, which parasitize reptiles [292]. Laboratory observations of that sort involve a non-natural ant diet in an artificial environment that might affect ant food preferences. Castellanos et al. [280] found that *S. invicta* was associated with reduced ixodid populations in South Texas because *S. invicta* drove small vertebrate hosts away from the study areas.

Yellow-billed and red-billed oxpecker birds (*Buphagus africanus* L. and *B. erythrorhynchus* Stanley, respectively) are relatively effective predators of ixodids, and other ectoparasites, of large African mammals, but their area-wide impacts on ixodid populations have not been reported [293]. Unfortunately, oxpecker populations have declined in response to wild mammal reductions, toxic effects of broad-spectrum acaricides, and, perhaps, acaricide-related reductions of ixodids on cattle [294,295,296,297].

The mud flat fiddler crab, *Uca rapax* (Smith), which consumes ixodid eggs on the South Texas coastal plains, is the only predator that has been shown to reduce ixodid populations [283,291]. Substantial year-round numbers of *U. rapax* occur on areas of saline soil, and the crabs feed on *A. americanum* eggs under controlled and natural field conditions [269]. Approximately 80% of *A. americanum* egg masses on the soil surface were eliminated overnight, suggesting that, because ixodid eggs generally require ≥2 w to hatch (e.g., *A. americanum* eggs take 31–60 d to hatch [298] and southern cattle fever tick, *Rhipicephalus (Boophilus) microplus* (Canestrini) eggs take 14–46 d [299,300], *U. rapax* elimination of ixodid egg masses might approach 100% before hatching can occur [269]. *Uca rapax* populations, numbers of crab tunnel entrances, and egg removal activity are relatively stable across all four seasons in saline habitats [269]. An apparent hatch of *U. rapax* resulted in a sudden June population peak, when ixodid egg predation likely intensifies [269]. Heavy sea ox eye daisy, *Borrichia frutescens* (L.) DC, stands, typical of the saline habitats, were mapped using global information systems technology, and comprised ≈24% of the wildlife corridor where ixodid populations are negligible compared to relatively low-salinity areas (where *U. rapax* is functionally absent) [269]. Limited areas (<1%) of saline habitats on the South Texas coastal plain, however, are matted by low-growing shoregrass, *Monanthochloe littoralis* Engelm., that appear to impede *U. rapax* foraging, thereby protecting ixodid eggs [269].

### 7.2. Parasitoids

Ixodids are utilized by various parasitoid encyrtid wasps, although they are not known to appreciably reduce ixodid populations [301]. The parasitoids *Ixodiphagus hookeri* Howard and *I. texanus* Howard occur in the United States [301,302], and there are additional related species that occur in other parts of the world [303,304,305,306,307,308,309,310]. Blood-engorged ixodid larvae are most commonly parasitized, but parasitoid eggs can also be retained through the larval molt to the nymphal stage where they complete their development to adulthood in engorged nymphs [301,305]. Typically, *Ixodiphagus* wasps lay 6–50 eggs within each host (some host ixodids are superparasitized, increasing the number of eggs they hold [301], and larger ixodids often receive more eggs than smaller hosts [310,311,312,313,314]; the parasitoids undergo development inside the ixodid host in ≈45 d [304]. Searching behavior is not well understood, and it is uncertain whether the parasitoids attack ixodid larvae while on- or off-host [301]. Because the wasps are not strong fliers, their ability to actively seek ixodid hosts on livestock and wildlife might be weak [301,315].

Up to 40% of nymphal blacklegged ticks, *Ixodes scapularis* (Say), have been parasitized [312,313,316], and Lyon et al. [315] indicated that the average parasitization rate is ≈23%. In Africa and India, rates of parasitism on *A. variegatum* and the bont-legged tick, *Hyalomma anatolicum anatolicum* Koel were >50% [308,311], but that is insufficient for achieving ixodid control [301]. Releases of ixodid parasitoid wasps [306,317,318,319] have mostly failed to exert control [301,302]. Knipling [320] suggested that parasitoid wasps must be released in substantial augmentative quantities to be effective in an ixodid management role. The only feasible way of accomplishing that is by mass-rearing the parasitoids [301,320] on ixodids maintained on living vertebrate hosts.

### 7.3. Entomophagous Nematodes

Considered to be environmentally benign biological control agents against ixodids [321,322,323,324], entomophagous nematodes have been tested on ixodids [92,325,326,327,328,329,330,331,332,333,334,335,336,337]. Samish et al. [338,339] reported that entomopathogenic nematodes can infect ≥16 ixodid species from six genera, and, in general, that heterorhabditids are more virulent than steinernematids. Various entomopathogenic nematode species have different degrees of virulence, and the species of ixodid affects host vulnerability [325,326,331,333,337,340,341,342,343,344]. Entomophagous nematodes *Steinernema riobravis* (Cabanillas & Poinar) [345] and *Steinernema feltiae* (Filipjev) invaded 96%, 89%, 24%, and 88% of replete female American dog ticks, *Dermacentor variabilis* (Say); brown dog ticks, *Rhipicephalus sanguineus* Latreille; *A. maculatum*; and the Cayenne tick, *Amblyomma cajennnense* (F.), respectively [327]. Although some nematodes enter ixodids, they do not produce a generation of infective juveniles, and the invading nematodes are isolated from surrounding tissues by a vacant space, suggesting that the host ixodid might produce defensive chemicals [327,342]. Fully engorged cattle fever ticks, *Rhipicephalus (Boophilus) annulatus* (Say), were vulnerable to steinernematid and heterorhabditid entomophagous nematodes [325,326,341,346], but *Steinernema carpocapsae* (Weiser) and *Steinernema glaseri* (Steiner) were unable to infect *A. variegatum* and *R. microplus* [326] despite being infective against *I. scapularis* [342]. *Amblyomma americanum* was killed by *S. glaseri*, *S. riobbravus*, *S. carpocapsae*, *S. feltiae*, and *H. bacteriophora* [327], while engorged immature *A. cajennense* mortality was only 13% after exposure to infective *S. glaseri* juveniles [347].

*Steinernema carpocapsae*, *S. glaseri*, *S. feltiea*, *Steinernema ceratophorum* (n. sp.), and *Heterorhabditis bacteriophora* Poinar, albeit lethal to *Dermacentor silvarum* Olenev, had different potencies in terms of inducing mortality and reduced egg deposition [344]. As another example, *S. carpocapsae* caused 100% mortality to *R. annulatus* females within 8 d [325,341], but *I. scapularis* mortality did not reach 100% until 17 d after infection [342]. The most susceptible sex and condition of ixodid hosts is engorged females (in contrast to less susceptible nonfed and partially fed females), but nonfed males are also killed [344].

Entering the ixodid host though a variety of orifices on the ixodid body [326,327,341,342,348,349,350,351,352,353,354], entomophagous nematodes normally reproduce inside, producing thousands of juvenile nematodes that emerge ≈2–3 w later to infect additional hosts [322,325,341,355]. In most instances, the efficacy of entomophagous nematodes is dose-dependent, and at higher doses, ixodid mortality often reaches a plateau [344]. Singh et al. [337] reported that, although *Heterorhabditis* and *Steinernema* species caused different levels of mortality against *R. microplus*, only immersion time had an effect on reproductive potential.

While some researchers have reported that entomophagous nematodes produce infective juvenile nematodes inside ixodid hosts that attack other ixodids [322,355,356], other reports indicate that *Xenorhabdus* bacteria, which are symbiotic with the infective nematodes [357,358,359], multiply to lethal numbers within the ixodid host (Kocan et al. 1998), causing septicemia often by 24–48 h [360], and the bacteria produces protective antibiotic-like chemicals that inhibit growth of other microorganisms [360,361].

Entomophagous nematodes are most commonly found in soil and are most likely to infect engorged female ixodids in particular, because they fall from the host upon the soil surface [322]. In the soil, survival of the infective juveniles is influenced by moisture, temperature, and soil composition and chemistry 325,331,338,342, (. Samish et al. [266] found that ixodid exposure to entomophagous nematodes must occur for extended periods of time, up to 32 h, to attain the greatest levels of control. Entomopathogenic nematode efficiency, however, was reduced when soil moisture declined below 8% [362], and when sandy soil was amended to 25% cattle manure or 40–50% silt [338]. Juveniles orient to hosts in response to chemotactic cues [338,363,364] that are influenced by soil type and chemistry. Application of nematodes for ixodid control will, in many instances, be limited to specific seasons, particularly in the northeastern United States where low temperatures can be lethal [342]. In addition, the nematodes’ moisture requirement will impede their efficacy in dry seasons and habitats.

Some protection from insufficient moisture was gained by formulating *H. bacteriophora*, *S. carpocapsae*, and *Steinernema websteri* (n sp.) in oil suspensions of 13% lemongrass, *Cymbopogon citratus* (de Candolle) Stapf, and Virginia juniper, *Juniperus virginiana* L. [365]. The oils maintained nematode survival at 55% to 60% for 96 h under laboratory conditions, and 33% oils were 80% to 100% effective for 24 h [365]. *Steinernema websteri* juveniles in the *J. virginiana* oil emulsion killed 90% of *I. scapularis* on dogs [365]. Others suggested that infective *Heterorhabditis floridensis* Nguyen, Gozel, Koppenhöfer, and Adams (K22 strain) juveniles will be effective for *R. microplus* control when applied in cattle dipping vats [337]. The commercial field utility of nematodes for ixodid control, however, has yet to be demonstrated [332].

A filarial nematode, *Yatesia hydrochaerus* Yates and Jorgenson, commonly found in capybaras, *Hydrochoerus hydrochaeris* L., was obtained from inside *A. cajennense* and *A. americanum* [366]. Metagenomic research indicated that *I. scapularis* is also infected by a filarial *Monanema* nematode, but its occurrence in ixodids has not been shown to reduce the ability of the ixodid to carry and transmit *B. burgdorferi* [367]. Similarly, *Monanema*-like DNA was found in ixodids in parts of the United States [368]. Other ixodids have been parasitized by *Acanthocheilonema* filarial nematodes [369], but filarial nematodes have not been applied for ixodid management.

### 7.4. Entomopathogens

A number of entomopathogenic microorganisms cause mortality to, and have sublethal effects on, ixodids. Although viruses and virus-like particles occur in ixodids without apparent negative effects [370], a rickettsia that caused epidemic typhus, *Rickettsia prowazekii* Katsinyian, was lethal against Rocky Mountain wood ticks, *Dermacentor andersoni* Stiles; the ornate sheep tick, *Dermacentor marginatus* Sulzer; and *Dermacentor reticulatus* (F.), but it did not adversely affect the camel tick, *Hyalomma dromedarii* Koch, and *Hyalomma anatolicum excavatum* Koch [266,371]. Rickettsia-like organisms occur in nearly all ixodid species mostly as symbiotes [372], but a rickettsia-like organism caused up to 50% mortality against engorged female *Rhipicephalus bursa* Canestrini and Fanzago. Kurtti et al. [373] found rDNA in *Ixodes woodi* Bishopp indicative of an endosymbiotic rickettsia-like organism. Microbial organisms that infect ixodids and cause adverse responses are described below.

#### 7.4.1. Bacteria

Ixodids have been associated with many species of bacteria that are apparently benign to the host [328,329,372]. As many as 73 bacterial isolates were identified on wild-caught *I. scapularis* [374]. Machado-Ferreira et al. [375] reported 17 types of bacteria, including *Staphylococcus* spp. and *Pseudomonas* sp., associated with *A. cajennense* eggs. A number of bacteria, however, are pathogenic to ixodids, such as *Proteus mirabilis* Hauser to *D. andersoni* [376], the South African bont tick, *Amblyomma hebraeum* Koch; the Mediterranean hyalomma tick, *Hyalomma marginatum* Koch; the red-legged tick, *Rhipicephalus evertsi-evertsi* (Neumann) [377]; the African blue tick, *Rhipicephalus (Boophilus) decoloratus* Koch [293], and *R. microplus* [378,379,380]. Under laboratory conditions, *Cedecia lapagei* (Enterobacteriaceae) can kill 100% of *R. microplus* and halt egg production [378,379,380].

*Bacillus thuringiensis* Berliner is the most widely recognized acaricidal bacteria. In laboratory studies, *B. thuringiensis* varieties (i.e., *kurstaki*, *israeliensis*, and *thuringiensis*) killed nonfed and engorged adult *H. dromedarii* [293]. *Bacillus thuringiensis kurstaki* also caused high mortality in adult female *R. microplus* [381]. Four strains of *B. thuringiensis* were equally toxic to adult female *R. microplus* in the laboratory [382]. The eggs of *H. dromedarii* were also susceptible to *B. thuringiensis*, and 96% mortality was achieved against engorged *I. scapularis* larvae [383,384].

Pathogenic bacteria penetrate ixodids through several portals. *Cedea lapagei*, for example, enters by the genital opening (e.g., in *R. microplus*) [378,379,380]. Although *B. thuringiensis* is ingested by insects and the microorganism infects the midgut, ixodids feed on blood directly from the host. A hygroscopic ixodid secretion that absorbs ambient moisture from the air is reingested by the ixodid, hence, it is possible that contact with *B. thuringiensis* solutions results in ingested bacteria [293]. It is also possible that *B. thuringiensis* exotoxins kill ixodids [385], the bacteria damage the hemocoele [386], and they occlude ixodid spiracles [293].

*Bacillus thuringiensis* produces crystalline δ-endotoxin during sporulation, which disrupts insect midgut walls [387]. Habeeb and El-Hag [388] reported that the 43-kDa Cry4Ba toxin of *B. thuringiensis* was highly toxic to engorged female *H. dromedarii* within 48 h by injuring cell membranes and granulocytes of the hemolymph, impeding the immune system. Sublethal effects have been associated with bacterial infection; *P. mirabilis* causes defects and mortality in the next generation of ixodid offspring [376].

#### 7.4.2. Protozoa

Few protozoans have been reported to kill ixodids, although several, including *Nosema* spp., occur in ixodids [266,389]. *Nosema slovaca* Weiser & Rehacek is pathogenic to ixodids, and *Hemolivia mauritanica* Sergent & Sergent is 50% lethal against the ixodid *Hyalomma syriacum* Koch [390].

#### 7.4.3. Fungi

Although more than 700 species of fungi are entomopathogenic, mostly in the classes Deuteromycetes and Eumycetes, only ≈20 species have been found in association with ≈13 ixodid species [293,383,391,392,393,394,395,396,397]. The most widely tested entompathogenic fungal genera, *Beauveria* and *Metarhizium*, have global distributions [398], both include multiple species, and the species have different genetic strains [397,399]. Fungi have likely been the most extensively investigated entomopathogens, with, for example, ≥30 studies on the lethal effects of *Metarhizium anisopliae* (Metschnikoff) Sorokin against *R. microplus* [400,401]. *Metarhizium anisopliae* is also pathogenic to other ixodids, such as *I. scapularis* and *R. sanguineus* [293,402,403,404,405,406,407], and *Beauveria bassiana* (Bals.) Vuill. virulence was demonstrated against many species, including brown ear ticks, *Rhipicephalus appendiculatus* Neumann; *R. sanguineus*; *R. decoloratus*; *Hyalomma* spp.; *A. americanum*; and *A*, *variegatum* [399,408,409,410,411,412,413].

Only 7.5% of wild caught adult castor bean ticks, *Ixodes ricinus* (L.), were infected by fungi in the winter, and 50% were infected during the summer [394]. Kalsbeek et al. [414] also found that natural fungal infections of engorged *I. ricinus* females were influenced by season. Other reports indicate that *R. appendiculatus* larvae in grassy livestock paddocks sprayed monthly with *B. bassiana* or *M. anisopliae* were not affected during the rainy season, but three months after the rainy season ended, the larval populations declined by 80% and 92%, respectively [415,416]. While many researchers have documented the effects of entomopathogenic fungi on ixodids, successes in the field have been relatively limited, weak, and inconsistent [417]. In addition, ixodid life stages in natural conditions have different susceptibilities to some fungi whereby adults were more infected than preimaginal stages [383,414]. Samish et al. [293] reported that, under laboratory conditions, ixodids show high mortality from naturally occurring fungi, but mortality in the field is generally low. This is verified by low recoveries of entompathogenic fungal species on ixodids in natural conditions [418,419]. *Metarhizium anisopliae* sprayed on a plantation infected 57% of *I. ricinus* [293], and Benjamin et al. [406] reported that a commercial formulation of *M. anisopliae* (Bio-Blast Biological Termicide), used for termite control, applied on questing *I. scapularis* adults killed 53% within 4 w. Kaaya et al. [409] reported that, under field conditions, *M. anisopliae* caused only 30–37% kill against *A. variegatum* and *R. appendiculatus*. Similarly, 36.4% of *I. scapularis* nymphs were infected following field applications of *M. anisopliae* [420,421], and Hornbostel et al. [422] indicated that, in the field, *M. anisopliae* did not appreciably affect numbers of questing *I. scapularis* nymphs. Other researchers reported that *B. bassiana* induced only 18–32% mortality against field populations of *R. microplus* [423], and that *B. bassiana* provided, at most, 58.7% field control of *I. scapularis* the field [421]. Alternatively, two commercial *B. bassiana*-based products registered for ornamental and turf pest control reduced nymphal *I. scapularis* by up to 89% [424].

Entomopathogenic fungi can kill ixodids while they are on living hosts [417]. Numbers of nymphal *R. sanguineus* dropping from gerbil hosts decreased by 73% after treatment with *M. anisopliae* [293]. While different life stages of *R. microplus* and *R. decoloratus* on cattle were not killed in appreciable numbers (≤50%) from a *M. anisopliae* spray, up to 79% of adult females obtained from the hosts died and egg mass weight declined by up to 50% [402,403,404,405]. Treating cattle with *M. anisopliae* spores killed 83% of adult *R. appendiculatus* [409]. On the other hand, treatment of white-footed mouse, *Peromyscus leucopus* (Rafinesque), nests with *M. anisopliae* resulted in modest ixodid population reductions [425]. Variability is exemplified by the negligible effect of *M. anisopliae* against *R. microplus* on cattle [403] versus a report of 50% reduction [402]. On the other hand, Rijo [426] reported that repeated applications of *B. bassiana* on cattle reduced ixodids by up to 93.5%.

Although most fungal infections of ixodids occur by direct contract, Suleiman et al. [427] suggested that transovarian transmission is also possible. Fungal entomopathogens, such as *M. anisopliae*, typically weaken the cuticle with histolytic enzymes [333,427], which sometimes ruptures, and mortality occurs as infected areas of the cuticle enlarge over ≥50% of the body surface area [412,428,429,430]. Lethal expansion of weakened parts of the cuticle can be relatively rapid, within 48 h under optimal ambient conditions [430]. Symptoms of fungal infection include changes in reproductive rate, decreased sensitivity, loss of appendage coordination, and paralysis, followed by death [401,431]. Although destruxin produced by *M. anisopliae* has been claimed to cause death in ixodids [432], Gôlo et al. [433] observed no effect when the compound was applied to *R. microplus*. Fungal growth continues in the ixodid cadaver, followed by sporulation [401,430]. Similarly, *B. bassiana* produces cuticle-degrading hydrolytic extracellular enzymes that enhance its virulence, allowing hyphae to penetrate the cuticle and invade the ixodid [423,434].

Different fungal species are more pathogenic than others, whereby *M. anisopliae* and *B. bassiana* are generally more virulent than other known infective species [339,435,436], and *M. anisopliae* is more virulent than *B. bassiana* [405,437,438,439,440,441], including expression of sublethal effects [442]. *Metarhizium brunneum* Petch is another fungal species that has shown notable virulence against as many as three ixodid species (*R. annulatus*, *R. sanguineus*, and *Hyalomma excavatum*) [339,436,443], and different strains and isolates of fungal entomopathogens, such as *M. anisoplieae* and *B. bassiana*, are differently virulent [411,429,430,444].

Susceptibility to fungal entomopathogens is also affected by the species of the tick, with some showing greater resistance than others [330,410,445]. Ostfeld et al. [397] suggested that *Ixodes* spp. were more vulnerable than *Rhipicephalus* (*Boophilus*) spp., and responses among *Amblyomma* spp. and other *Rhipicephalus* spp. were variable. While *B. bassiana* killed 100% of *A. americanum*, for example, mortality was not observed against *D. variabilis* [410].

The life stage of ixodids, including eggs, have different vulnerabilities to entomopathogenic fungi [293,330,339,413,415,436,439,445,446,447,448,449,450], with adults, in general, being more susceptible than larvae and nymphs [397]. Nonfed *R. appendiculatus*, *A. variegatum*, *H. excavatum*, and *R. sanguineus* showed decreasing susceptibility to fungal entomopathogens from larval to nymphal to adult life stages [339,414]. *Beauveria bassiana* application on potted grasses kept under natural conditions caused 96% and 37% mortality to *R. appendiculatus* nymphs and adults, respectively, and *M. anisopliae* killed 76% and 64%, respectively [293]. Conversely, nonfed *I. scapularis* larvae were not as susceptible as nonfed adults [383].

Another factor affecting entomopathogenic fungal virulence involves the ixodid’s feeding status, whereby engorging and engorged ixodids are reportedly more susceptible than nonfed individuals [397]. Nonfed larval and nymphal stages were less susceptible to *M. anisopliae* than engorged individuals [451]. On the other hand, engorged larval *H. excavatum* and *R. sanguineus* survived two times longer than nonfed larvae [293].

Fungal dosage, or exposure levels, further affect efficacy. Mortality is commonly reported as being dose-dependent [330,430].

In addition to inducing ixodid mortality, entomopathogenic fungi can induce sublethal effects. Infection of engorged females is sometimes associated with extended periods of preoviposition and oviposition, and egg incubation and hatching, and reduced egg production [339,417,436,440,447,452,453]. As an example, Kaaya et al. [409] demonstrated that eggs produced by *B. bassiana*-treated *R. appendiculatus* feeding on rabbits failed to hatch, and, on cattle 48% hatched (this suggests, too, that the vertebrate host species can influence sublethal effects). A laboratory and field study determined that *M. anisopliae* reduced *I. scapularis* egg production by up to 50% [453]. In addition, *M. anisopliae*-infected *I. scapularis* larvae and nymphs molted into their next life stages with weight reductions [397]; overall, fungal infection results in decreased ixodid fitness for survival [397]. Reduced egg production and hatching occurred after engorged *R. microplus* adults were treated with a commercial *Metarhizium*-based product [454], and *M. anisopliae* applied on female *H. anatolicum* reduced numbers of deposited eggs, hatching, and percent of molting offspring [427]. Sun et al. [399] reported that *B. bassiana* applied to engorged female *R. microplus* reduced oviposition, although most of the females died before laying eggs.

Aside from *B. bassiana* and *M. anisopliae*, other fungi infect ixodids, such as *Verticillium lecanii* (Zimmerman) Viegas [411], which, sprayed on cattle four times, reduced *B. microplus* infestations by up to 99% [455]. In addition, *Aspergillus ochraceus* Wilhelm, *Paecilomyces* spp., and *Lecanicillium* spp. were found on field-collected *I. scapularis* [383,418,456,457,458,459].

While entomopathogenic fungi can be acaricidal, they have several disadvantages. They require relatively high humidity to germinate and sporulate, high and low temperatures can degrade performance, they are substantially slower to cause mortality than some other tactics (e.g., conventional synthetic acaricides, botanical toxins, and desiccant dusts), the fungi are vulnerable to solar ultraviolet radiation, some entomopathogenic fungi have adverse effects against nontarget arthropods, mass production can be expensive [293,397,417,460,461,462,463,464,465,466,467,468,469,470], the lethal action can be relatively short [402,404,409], and some have been associated with disease in immunocompromised humans [471,472]. Samish et al. [293] suggested that low ixodid field mortality (even with a high incidence of fungi adhering to them, causing substantial laboratory mortality) likely results from poor germination and hyphal penetration, and sublethal infections.

Some disadvantages of fungi can be countered using amenable formulations compatible with conventional application methods [473]. Infective fungal spores (conidia) are commercially available in formulations, developed, in part, to mitigate deleterious effects of adverse environmental conditions [401,470,474,475,476,477]. The formulations can also increase virulence; for example, oil-based formulations tend to be more effective than aqueous formulations [397,478,479,480,481,482]. The addition of peanut oil (15%) to aqueous suspensions of *M. anisopliae* spores increased the organism’s lethality against *A. variegatum* nymphs and adults on vegetation by 30% and 2.7-fold, respectively [405]. The same formulation applied against *R. appendiculatus* was 15% and 4.2-fold, respectively, more effective than aqueous suspensions without peanut oil [405]. *Metarhizium anisopliae* conidia in an oil–water emulsion were strongly virulent to *R. microplus*, and the emulsion conferred some tolerance to elevated temperature [470]. On the other hand, Camargo et al. [454] reported variable efficacy of an oil-based commercial *M. anisopliae* product against *R. microplus* on cattle, and the average efficacy was <48%. Bharadwaj and Stafford [483] reported that emulsifiable concentrate and granular formulations of *M. brunneum* were effective against nonfed nymphal and adult *I. scapularis*. Moderate efficacy was also achieved using a polymerized cellulose gel carrier combined with surfactants, and sunscreens protected entomopathogenic fungal conidia against ultraviolet radiation [475,484,485]. Kaaya and Hassan [405] demonstrated that mixing *M. anisopliae* spores with millet-, corn-, sorghum-, and starch-based powders caused 100%, 79%, 64%, and 53% mortality against *R. appendiculatus* adults feeding on cattle. Fungal entomopathogens combined with conventional insecticides [480,486] and botanically-based extracts (e.g., chinaberry, *Melia azedarach* L. extract) [487], have shown efficacy against ixodids. Hornbostel et al. [422], for example, demonstrated that *M. anisopliae* mixed with permethrin did not mitigate the lethal activity of the fungus against *I. scapularis*. Further, mixtures of different fungal species, such as *B. bassiana* + *M. anisopliae*, and combinations of different strains of *M. anisopliae*, were more effective than either species and strains applied alone [486,488].

While entomopathogenic fungi might be useful for localized control of ixodids off- and on-host, it is possible that they could induce epizootic outbreaks [489] against area-wide ixodid populations. Bharadwaj and Stafford [420] suggested that, because ixodids spend most of their lives off-host on moist soil and organic debris [490], which are suitable for reproduction of fungi, entomopathogenic fungi represent an ixodid control tactic with strong potential.

## 8. Inert Dusts

Kaolin is a porous, fine-grained aluminosilicate white mineral clay that causes a variety of detrimental effects to arthropods [28]. Kaolin particles adhere to the arthropod integument, occluding the normal range of motion of appendages, strongly obstructing mobility, and they can also abrade the integumentary cuticle, leading to desiccation [491,492]. A commercial kaolin-based product, Surround WP (Engelhard, Iselin, NJ), with surfactants, disrupts the larval ixodid cuticle, resulting in relatively rapid desiccation (compared to nymphs) because the small larval body size is associated with a high evaporative surface area: volume ratio [28,493].

Surround WP was moderately lethal to *A. americanum* larvae and nymphs, but to a lesser extent than silica- and diatomaceous earth-based dusts [28,30]. On the other hand, larval and nymphal *A. americanum* exposed to aqueous suspensions dried on a surface resulted in higher larval (not nymphal) mortality than a dried aqueous suspension of a silica gel-based product (CimeXa) [28]. The greatest mortality, ≈90%, occurred when larvae crawled across dry Surround WP treated substrate, and nymphal mortality was as high as 70% [28].

An assessment of a variety of desiccant dust materials demonstrated that silica gel had the greatest insecticidal efficacy [494]. Dri-Die (Fairfield American, North Rutherford, NJ, USA) and CimeXa (Rockwell Labs, Kansas City, MO, USA) are composed of fine silica gel powders that adsorb ixodid cuticular wax into a matrix of pores between the dust particles [495,496,497,498]. Deterioration of the cuticular wax facilitates fatal dehydration [28].

Application of dry silica gel-based desiccant dusts have been effective against spinose ear ticks, *Otobius megnini* Duges), *A. americanum*, and *R. sanguineus* [28,29,494,499]. Brief immersion of *A. americanum* larvae and nymphs in dry silica gel (CimeXa) dust caused 100% mortality within 24 h and crawling ≈7.5 cm across filter paper with a thin film of the dust also resulted in complete mortality to both life stages by 24 h [28]. When *A. americanum* larvae and nymphs crawled across a thin film of dried aqueous silica gel (CimeXa) suspension, however, only ≈35% and ≈2%, respectively, died [28].

While *I. scapularis* mortality strongly declined when relative humidity was >81% [493], silica gel (CimeXa) applied to Gulf cordgrass, *Spartina spartinae* (Trin.) Merr. ex Hitchc., in 18–23 kph winds and 73.6% relative humidity killed >94% of questing larval and nymphal *A. maculatum* populations within 24 h [28]. The same product prevented larval *A. americanum* from feeding on stanchioned calves, suggesting that silica gel provides long-residual prophylactic protection of cattle from ixodid larvae [29]. Because *Rhipicephalus* (*Boophilus*) spp., which transmit the deadly agents that cause babesiosis in cattle, are one-host ixodids whereby only the larval stage quests, silica gel desiccant dusts are likely to be particularly effective against them [29].

Perlite is an amorphous absorbent [500] aluminosilicate volcanic rock material [501] often added to soil to conserve water and improve plant growth [502,503]. It is commercially available (Imergard WP (100% perlite), Imerys Filtration Minerals, Lompoc, CA, USA) for pest management purposes as a desiccant dust, but it has only been tested against ixodids (ATS, unpublished data). One day after crawling ≈7.5 cm across a thin layer of perlite dust, *A. americanum* larval mortality was 100% (ATS, unpublished data). Mortality was complete by 24 h in response to perlite and CimeXa treatments (ATS, unpublished data).

Diatomaceous earth-based dusts are natural silica products comprised of fossilized diatoms [504,505]. Efficacy of diatomaceous earths against arthropods vary depending upon composition [506,507,508]. Lethal desiccation is induced as by silica gel [505,508]. Larval and nymphal *A. americanum* that crawled ≈7.5 cm across a thin layer of the diatomaceous earth product, Deadzone (Imerys Filtration Minerals, Lompoc, CA), were killed at the same rate achieved by silica gel (CimeXa) [30]. A dried aqueous suspension of Deadzone, upon which larval and nymphal *A. americanum* crawled ≈7.5 cm, was less lethal than when the product was applied as a dry dust [30].

Desiccant dusts offer multiple and unique advantages not commonly available in conventional acaricides. Resistance to silica gel (and other desiccant dusts), in the form of thickened cuticle, is only known to have occurred in one Australian laboratory strain of bed bugs, *Cimex lectualrius* L. [509,510]. Desiccant dusts are not likely to induce the development of resistance in ixodids at least in part because treatment of vegetation and host animals will be intermittent, and the dusts can be rotated with chemically-based acaricides, in addition to desiccant dusts combined with chemically-based toxins [31,32]. In addition, the dusts might be amenable for organic animal production as well as in wildlife refuges, parks, and other protected areas where application of synthetic chemical pesticides is generally discouraged. Desiccant dusts, being nontoxic to vertebrates, pose a low risk to human applicators. Further, because they are inert and nonvolatile, inert dust efficacies can persist despite aging and exposure to sunlight and heat, and problems that might emerge from leaching into the soil and runoff have not been reported. The extended residual efficacy of inert desiccant dusts [511] can confer relatively long term prophylactic protection to livestock and other animals, particularly against larvae, before ixodids commence feeding [29]. Stability in the environment will likely reduce the need for repetitive interventions that typify use of chemically-based acaricides; hypothetically, desiccant dusts can persist in efficacious quantities until the particles are physically removed [28]. Although moderate rainfall and wind remove some dust particles on plant surfaces, residual dust remains visible [512]. Desiccant dusts (diatomaceous earth, perlite, and silica gel) are lethal to immature *A. americanum* and *A. maculatum*, and it is likely that they will also kill larvae and nymphs of additional ixodids. It is also possible that the dusts will control other arthropod ectoparasites of livestock and other vertebrates, including cattle louse species [494] and the horn fly, *Haematobia irritans irritans* (L.) (ATS, unpublished data). Another advantage of inert dusts involves indefinite shelf lives, including under poor storage conditions (e.g., heat, ultraviolet radiation, light, and aging). The dusts are amenable to augmentation with toxic chemicals, including botanical substances, that are acutely lethal to ixodids [29,31,32]. Moreover, desiccant dusts can be applied to livestock by active and passive means; active treatment involves equipment, such as manual and motorized “dusters” [28,513], while passive application involves, for example, dust bags that are commonly used for protecting cattle against ectoparasites [368,514,515]. Dusts might also be applied as bands or strips on vegetation so that host animals can pass through them, treating their legs in the process [516].

Potential disadvantages of inert dusts include slow action relative to conventional toxin-based acaricides [517,518]; the dusts, however, can result in complete mortality of larval ixodids within hours [28,29,30,31]. Dusts are less effective after being wetted and they are best applied to dry surfaces. Silica gel was ineffective against larval and nymphal ixodids while ingesting blood because the desiccant effect is offset by intake of host fluids [29].

Inert dusts can negatively affect nontarget arthropods. Surround WP, applied to crop systems, reduced numbers of cicadellids, some dipterans, and predators, including minute pirate bugs, *Orius* spp.; wasps [519]; green lacewings, *Chrysoperla carnea* (Stephens) [520]; earwigs; predatory mites; ladybird beetles; and spiders [521,522].

A commercial silica gel + pyrethrins product, Drione (Aventis, Montvale, NJ), is 40% (by weight) silica dioxide, 10% piperonyl butoxide (PBO), and 1% pyrethrins. Despite reports of ixodid resistance to pyrethroids [37,208], development of resistance to Drione is unlikely because it has physical (i.e., desiccation) and chemical (i.e., nerve toxin) modes of action [32,204,205]. Additionally, PBO is a synergist present in ≥1600 registered pesticides [523]. It renders arthropods temporarily susceptible to a variety of toxic chemicals that would otherwise, at the dose received, be nonlethal [523,524]. In effect, it makes resistant insects [524,525] and ixodids [526] susceptible. While *C. lectularius* has developed resistance to pyrethroid insecticides, Drione was effective against a resistant strain due to PBO, as well as the desiccating action of silica gel [510]. In laboratory bioassays, Drione killed >94% of *I. scapularis* larvae within 4 h [493]. Nymphal *I. scapularis* mortality in response to Drione was equivalent to that observed when using chlorpyrifos, a chlorinated organophosphate nerve toxin [493]. Brief immersion in dry Drione and exposure by crawling ≈7.5 cm over a thin layer of the product induced 100% mortality of larval and nymphal *A. americanum* within 2 and 4 h, respectively [32]. Aqueous Drione suspensions dried onto substrate were not as efficient because the product caked to the surface such that the silica particles were less available for adhering to the immature ixodids, and possibly because the pyrethrins were diluted in the aqueous suspension before it dried by evaporation [32]. Drione killed *A. americanum* larvae and nymphs on calves before they could begin feeding, and while they ingested blood because of the pyrethrins [29]. The silica gel component will continue to prophylactically protect animals from ixodids, even after the potency of the pyrethrins degrade, until the dust is physically removed from the animal [29].

EcoVia wettable dust (Rockwell Labs, North Kansas City, MO, USA) involves two active ingredients: silica gel (≈80%) and thyme, *Thymus vulgaris* L., oil (10%) [31]. Brief immersion in dry EcoVia dust and exposure by crawling over ≈7.5 cm of a thin layer of EcoVia on filter paper resulted in 100% mortality against larval and nymphal *A. americanum* within 1 and 2 h, respectively [31]. Similar to Drione, EcoVia probably eliminates nonfeeding and feeding larval and nymphal ixodids in response to the thyme oil’s toxicity, and it will also confer long-lasting prophylactic protection of animals and continue killing ixodids on vegetation due to the desiccating action of the silica gel [31]. EcoVia’s potency (similar to Drione’s) declined when the immature ixodids were exposed to a dried aqueous suspension [31].

## 9. Cultural Control Tactics

Ixodids require specific environmental conditions that protect them against desiccation and maintain humidity levels adequate for their survival and reproduction [527]. Nonchemical and nonbiological control tactics, such as habitat modification, controlled burning, and fenced enclosures can inhibit and reduce ixodid survival [528]. Nonmaintained grasslands and forests, for example, can accumulate detritus on the soil surface, promoting elevated soil level humidity that favors ixodid survival and reproduction [527]. Management of these areas, with the aim of ixodid control through the removal of leaf litter and decaying vegetation, will help limit the amount of ground-level debris. Alternative ground cover options, including mulch and stone, can reduce humidity, negatively affecting ixodid survival [529]. Creating walkways and maintained paths that inhibit elevated humidity also offer promising methods for suppressing ixodids by removing soil surface debris [530,531].

Reduction of soil debris, and low-growing foliage upon which ixodids quest for hosts, can also be achieved through controlled burns that clear large areas of vegetation-based ground cover [532]. Studies on the effects of burning on *Amblyomma* spp. indicated a 66% reduction in ixodid abundances across all life stages [532,533,534]. Annual (or more frequent) burns might provide long-term suppression of ixodid abundances by the temporally systematic removal of grasses and soil surface detritus [534]. Ixodid population reductions, however, are not necessarily correlated with decreases in specific life stages [533,535]. For example, although burned areas harbor up to 98% fewer ixodids, surrounding nonburned areas did not have suppressed ixodid populations [534]. Some studies suggest that ixodid population reductions resulting from burning are temporary [536] because ixodids periodically find shelter in the lowest regions of vegetation (to rehydrate between questing times) [536], possibly protecting the pests from direct exposure to flames [536]. It is nevertheless conceivable that regular burning might hold ixodid numbers to relatively low, or inconsequential, levels. Regular burning might provide a potential solution for preventing substantial ixodid numbers from returning and, effectively, eliminate ixodids in formerly infested habitats.

Fenced enclosures present physical barriers that exclude wild hosts from mingling with livestock. The movement of deer, hosts to a wide range of ectoparasitic arthropods [537], into cattle pastures and rangeland can be restricted by fencing [516]. Deer exclusion from a fenced-off area in New York for 25 y reduced ixodid infestations by ≈90% on mammalian hosts [464,538]. In South Texas, fencing has prevented unrestricted movement of some nilgai populations [539]. Ineffective barriers, such as the three-stranded barbed wire fences that are typically used, can be permeable to wildlife, including deer and nilgai [516]. Enclosures are not feasible in all systems, but, where applicable, they contain large mammalian host movement, limiting the dissemination of ixodid into, and out of, the fenced areas. Manmade barriers, including fences, canals, and roads with nonporous center strips, have been suggested for containing white-tailed deer and nilgai populations that can be treated in isolations to eradicate existing populations of *Rhipicephalus* spp. ixodids that transmit the causal agents of bovine babesiosis [516].

## 10. Applied Prospects for Ixodid Management

Most alternative control tactics have not yet been adopted for ixodid management on localized and area-wide bases, and the majority of substances mentioned have not been developed for commercial use. As ixodid resistance to conventional acaricides increases, the demand and consequent search for alternatives are likely to intensify. As demand for rangeland and woodland ixodid control (e.g., eradication of cattle fever ticks from South Texas [540] intensifies, the need for applying the currently available environmentally disruptive acaricides in those habitats will also increase. Some alternative control tactics might be acceptable for use in protected habitats because they are naturally occurring, have negligible environmental impacts, do not accumulate in the trophic web, and they do not involve highly persistent toxins. Alternative control methods offer novel tools to augment the limited contemporary ixodid control arsenal as part of integrated pest management and eradication strategies. Some nonconventional tactics can be rotated with conventional tactics and used in combination with conventional tactics and with other alternative tactics, to heighten efficacy while impeding the development of resistance. Many alternative tactics are more lethal against some species, life stages, and feeding status (nonfed, feeding, and engorged) of ixodids than others, offering a broad range of tools specific to extant conditions, including some nonconventional substances that, instead of inducing mortality, negatively affect ixodid reproduction, and deter or repel ixodids away from feeding. Desiccant dusts might facilitate long-residual ixodid control on vegetation, livestock, and wildlife. The great array of biological control possibilities, growth regulators, plant-based substances, and inert dusts offer many possibilities that can be tailored for specific target ixodid species, life stages, habitats, and ambient conditions. The need for investigating alternative ixodid control tactics has not diminished; hence, the ability to select the best fit strategy for a range of circumstances is likely to improve.

## Figures and Tables

**Table 1:** Selected studies that report lethal and sublethal effects of botanical extracts (solvents, exposure times, experimental conditions, and other parameters are not included).

Plant Species	Ixodid Species	Effects ^a^	Citation
*Acacia nilotica*	*Rhipicephalus microplus*	Mortality	[68]
(gum Arabic tree)			
*Achyranthes aspera*	*Haemophysalis bispinosa*	Mortality	[69]
(chaff flower)	*R. microplus*	Mortality	[70]
*Acmella oleracea*	*R. microplus*	Mortality, inhibits reproduction	[71]
(paracress)			
*Acorus calamus*	*R. microplus*	Mortality, inhibits reproduction	[72]
(sweet flag)			
*Aegle marmelos*	*H. bispinosa*, *R. microplus*	Mortality	[73]
(Bengal quince)			
*Ageratum conyzoides*	*Amblyomma cajennense*	Repellency	[74]
(Mexican tea)			
*Allium sativaum*	*Rhipicephalus annulatus*	Mortality, inhibits reproduction	[75]
(garlic)			
*Andrographis lineata*	*R. microplus*	Mortality	[73]
(striped false waterwillow)			
*Andrographis paniculata*	*R. microplus*	Mortality	[73]
(green chiretta)			
*Anisomeles malabarica*	*H. bispinosa*	Mortality	[69]
(Malabar catmint)	*R. microplus*	Mortality	[70]
*Annona muricata*	*R. microplus*	Mortality, inhibits reproduction	[76]
(soursop)			[77]
*Annona squamosa*	*Hyalomma anatolicum*	Mortality, inhibits reproduction	[78]
(soursop, custard)	*H. bispinosa*, *R. microplus*	Mortality	[76]
*Artemisia annua*	*R. microplus*	Mortality	[79]
(sweet wormwood)			
*Azadirachta indica*	*Amblyomma americanum*	Mortality, deterrence	[80]
(neem)	*A. americanum*	inhibit reproduction	[81]
	*Amblyomma hebraeum*	Mortality	[82]
	*Hyalomma truncatum*		
	*Rhipicephalus evertsi*		
	*Dermacentor variabilis*	Inhibit reproduction	[83]
	*R. microplus*	Inhibit reproduction	[84]
	*Rhipicephalus sanguineus*		
	*R. microplus*	Mortality	[85]
	*R. microplus*	Mortality	[76]
	*R. microplus*	Mortality	[86]
	*R. microplus*	Inhibit reproduction	[87]
	*R. microplus*	Mortality, inhibit reproduction	[88]
*Brunfelsia uniflora*	*R. microplus*	Mortality	[89]
(manacá)			
*Buxus papillosa*	*R. microplus*	Mortality	[68]
(boxwood)			
*Calea serrata*	*R. microplus*	Mortality	[90]
(snake herb)	*R. sanguineus*		
*Callicarpa americana*	*A. cajennense*	Repellency	[74]
(beautyberry)			
*Callitropis procera*	*R. microplus*	Inhibits reproduction	[91]
(silk cotton)			
*Capsicum frutescens*	*R. microplus*	Inhibits reproduction	[92]
(tabasco pepper)			
*Cassia auriculata*	*R. microplus*	Mortality	[93]
(matura tea tree)			
*Cassia didymobotrya*	*Rhipicephalus appendiculatus*	Repellency	[94]
(candelabra tree)			
(golden shower)			
*Chenopodium ambrosioides*	*A. cajennense*	Repellency	[74]
(Jesuit’s tea)			
*Chrysanthemum*	*A. americanum*	Mortality	[29]
*cinerariaefolium*	*A. americanum*	Mortality	[32]
(chrysanthemum)	*R. sanguineus*	Mortality	[95]
*Cissus adenocucaulis*	*R. appendiculatus*	Repellency	[94]
(pink cissus)			
*Cocculus hirsutus*	*R. microplus*	Mortality	[73]
(broom creeper)			
*Copaifera reticulate*	*R. microplus*	Mortality	[96]
(copaiba balsam)			
*Cupressus nootkatensis*	*R. microplus*	Mortality	[97]
(Alaska yellow cedar)			
*Cymbopogon citratus*	*R. microplus*	Mortality	[98]
(lemongrass)	*R. microplus*	Inhibition of egg laying	[84]
	*R. microplus*	Mortality	[85]
*Dahlstedtia pentaphylla*	*R. microplus*	Mortality	[99]
(no common name)	*R. microplus*	Repellency	[100]
*Datura metel*	*R. microplus*	Mortality, inhibits reproduction	[101]
(Indian thornapple)			
*Eucalyptus globoidea*	*Hyalomma marginatum*	Repellency	[102]
(southern blue gum)			
*Eucalyptus* spp.	*R. microplus*	Mortality	[103]
*Euphorbia cyparissias*	*Ixodes ricinus*	Mortality	[104]
(cypress spurge)			
*Euphorbia prostrate*	*H. bispinosa*	Mortality	[105]
(prostrate spurge)			
*Euphorbia hirta*	*R. appendiculatus*	Repellency	[94]
(asthma plant)			
*Fumaria parviflora*	*R. microplus*	Mortality	[68]
(fineleaf fumitory)			
*Gloriosa superba*	*H. bispinosa*	Mortality	[69]
(flame lily)	*R. microplus*	Mortality	[70]
*Gynandropsis gynandra*	*Amblyomma variegatum*	Mortality	[106]
(Shona cabbage)	*Rhipicephalus appendiculatus*		
*Hypericyum polyanthemum*	*R. microplus*	Mortality, inhibit reproduction	[107]
(no common name)			
*Jatropha curcas*	*R. annulatus*	Mortality, inhibit reproduction	[108]
(physic nut)			
*Kigelia africana*	*R. appendiculatus*	Repellency	[94]
(sausage tree)			
*Leucaena leucocephala*	*R. microplus*	Mortality	[109]
(leucaena)			
*Lonchocarpus* spp.	*R. microplus*	Mortality	[110]
(rotenone)			
*Lysiloma latisiliquum*	*R. microplus*	Mortality	[109]
(false tamarind)			
*Magonia pubescens*	*R. microplus*	Mortality	[111]
(no common name)			
*Mammea siamensis*	*R. microplus*	Mortality	[112]
(salapee)			
*Matricaria chamomilla*	*R. annulatus*	Mortality, inhibit reproduction	[113]
(German chamomile)			
*Melia azadirach*	*A. cajennense*	Mortality	[74]
(chinaberry)	*R. microplus*	Mortality	[114]
	*R. microplus*	Mortality	[115]
	*R. microplus*	Mortality, inhibit reproduction	[116]
	*R. microplus*	Inhibit reproduction	[117]
*Melinus minutiflora*	*R. appendiculatus*	Repellency	[118]
(molasses grass)	*R. microplus*	Repellency	[119]
*Memora nodosa*	*A. cajennense*	Repellency	[74]
(no common name)			
*Mentha piperita*	*A. hebraeum*	Repellency	[120]
(peppermint)			
*Mentha pulegium*	*A. cajennense*	Repellency	[74]
(pennyroyal)			
*Neorautanenia mitis*	*R. appendiculatus*	Inhibits reproduction	[121]
(gemsbokboontjie)			
*Nigella sativa*	*Ixodes scapularis*	Repellency	[122]
(black cumin)	*R. annulatus*	Mortality	[123]
*Palicourea marcgravii*	*R. microplus*	Mortality	[124]
(no common name)			
*Pelargonium graveolens*	*A. americanum*	Repellency	[125]
(sweet-scented geranium)			
*Petiveria alliacea*	*R. microplus*	Mortality	[126]
(anamu)	*R. microplus*	Mortality, inhibit egg laying and hatch	[127]
*Piper adancum*	*R. microplus*	Mortality, inhibits reproduction	[128]
(spiked pepper)	*R. microplus*	Mortality, inhibits reproduction	[129]
*Piper tuberculatum*	*R. microplus*	Mortality, inhibits reproduction	[130]
(cordoncillo)	*R. microplus*	Mortality	[131]
*Piscidia piscipula*	*R. microplus*	Mortality	[109]
(Jamaican dogwood)			
*Psidium guajava*	*H. bispinosa*	Mortality	[69]
(guava)	*R. microplus*	Mortality	[70]
*Rhinocanthus nasutus*	*H. bispinosa*, *R. microplus*	Mortality	[93]
(snake jasmine)			
*Ricinus communis*	*R. microplus*	Mortality	[132]
(castor bean)	*R. microplus*	Mortality	[70]
	*R. sanguineus*	Mortality	[133]
*Ruta graveolens*	*Amblyomma cajennense*	Repellency	[74]
(common rue)			
*Senna italica*	*H. marginatum*	Mortality	[134]
(Port Royal senna)			
*Solanum trilobatum*	*H. bispinosa*	Mortality	[69]
(thoothuvalai)	*R. microplus*	Mortality	[70]
*Spiranthera odoratissima*	*A. cajennense*	Repellency	[74]
(no common name)			
*Stemona curtisii*	*R. microplus*	Mortality	[112]
(yan ling)			
*Stylosanthes humilis*	*R. microplus*	Repellency	[135]
and *S. hamata*	*R. microplus*	Mortality	[136]
(Townsville stylo and			
Caribbean stylo)			
*Szygium malaccensis*	*R. microplus*	Mortality	[85]
(Malay apple)	*R. microplus*	Mortality	[76]
*Tageta patula*	*R. sanguineus*	Mortality, inhibits reproduction	[137]
(French marigold)			
*Tamarindus indica*	*R. microplus*	Mortality	[138]
*Tephrosia vogelii*	*R. appendiculatus*	Mortality	[139]
(fish poison bean)	*R. appendiculatus*	Mortality	[140]
	Ixodids (spp. not reported)	Mortality	[141]
*Thymus vulgaris*	*A. americanum*	Mortality	[31]
(thyme)	*R. annulatus*	Mortality	[123]
	*R. microplus*	Mortality	[142]
	*R. sanguineus*	Mortality	[143]
*Tropaeolum majus*	*R. microplus*	Inhibits reproduction	[144]
(nasturtium)			
*Vachellia pennatula*	*R. microplus*	Mortality	[109]
(fern-leaf acacia)			
*Vitex agnus castus*	*Amblyomma* spp.	Repellency, detach from host	[145]
(lilac chastetree)	*I. ricinus*, *R. sanguineus*		
*Vitex negundo*	*H. bispinosa*, *R. microplus*	Mortality	[93]
(Chinese chastetree)			
*Withania somnifera*	*R. microplus*	Inhibits reproduction	[146,147]
(poison gooseberry)			

^a^ Inhibits reproduction includes negative effects on egg production, laying, and hatchability.

**Table 2:** Selected studies that report lethal and sublethal effects of essential oils (solvents, exposure times, experimental conditions, and other parameters are not included).

Plant Species	Ixodid Species	Effects ^a^	Citation
*Achillea millefolium*	*Ixodes ricinus*	Repellency	[148]
(yarrow)			
*Agathis ovata*	*Rhipicephalus microplus*	Mortality	[149]
(mountain kauri)			
*Ageratum houstonianum*	*Rhipicephalus lunulatius*	Mortality	[150,151]
(bluemink)			
*Alpinia zerumbet*	*R. microplus*	Mortality	[152]
(shell ginger)			
*Amyris balsamifera*	*Amblyomma americanum*,	Repellency	[153]
(amyris)	*Ixodes scapularis*		
*Annona squamosal*	*Hyalomma anatolicum*	Mortality, inhibits reproduction	[154]
(soursop, custard)	*R. microplus*		
*Artemisia annua*	*Rhipicephalus annulatus*	Mortality	[155]
(sweet wormwood)			
*Artemisia herba-alba*	*I. ricinus*	Repellency	[156]
(white wormwood)			
*Azadirachta indica*	*Amblyomma variegatum*	Mortality	[157]
(neem)	*Hyalomma. anatolicum*		
	*excavatum*	Mortality, inhibits reproduction	[158]
	*Hyalomma dromedarii*	Mortality, growth regulation	[159]
	*R. microplus*	Mortality, inhibits reproduction	[160]
*Baccharis dracunculifolia*	*R. microplus*	Mortality	[161]
(alecrim-do-campo)			
*Calea serrate*	*R. microplus*	Mortality	[162]
(snake herb)			
*Calendula officinalis*	*I. ricinus*	Repellency	[156]
(pot marigold)			
*Callitris sulcata*	*R. microplus*	Mortality	[149]
(sapin de camboui)			
*Calocedrus decurrens*	*I. scapularis*	Mortality	[163]
(incense cedar)			
*Carapa guianensis*	*R. microplus*	Mortality	[164]
(andiroba)	R. microplus	Mortality, inhibits reproduction	[165]
	*Rhipicephalus sanguineus*	Inhibits reproduction	[166]
	*R. sanguineus*	Mortality, inhibits reproduction	[167]
*Chamaecyparis lawsoniana*	*I. scapularis*	Mortality	[163]
(Port Orford cedar)			
*Chamaecyparis nootkatensis*	*I. scapularis*	Mortality	[97]
(Alaska yellow cedar)	*I. scapularis*	Mortality	[168]
	*I. scapularis*	Repellency	[169]
*Citrus limonum*	*R. microplus*	Mortality	[170]
(citrus lemon)			
*Citrus maxima*	*R. microplus*	Mortality	[171]
*Citrus reticulata*	*R. microplus*	Mortality	[112]
(mandarin orange)			
*Conyza dioscoridis*	*I. ricinus*	Repellency	[156]
(ploughman’s spikenard)			
*Curcuma longa*	*R. microplus*	Mortality	[112]
(turmeric)			
*Cuminum cyminum*	*R. microplus*	Mortality	[172]
(cumin)			
*Cunila angustifoli*	*R. microplus*	Mortality	[173]
(no common name)			
*Cunila incana*	*R. microplus*	Mortality	[173]
(no common name)			
*Cunila spicata*	*R. microplus*	Mortality	[173]
(no common name)			
*Curcuma zedoaria*	*Dermacentor nitens*	Mortality	[174]
(zedoary)			
*Cymbopogon citratus*	*R. microplus*	Inhibits reproduction	[175]
(lemon grass)			
*Cymbopogon martinii*	*R. microplus*	Mortality	[130]
(gingergrass)			
*Cymbopogon nardus*	*A. cajennense*, *Anocentor*	Mortality	[176]
(citronella grass)	*nitens*		
*Cymbopogon schoenanthus*	*R. microplus*	Mortality	[130]
(camel grass)			
*Cymbopogon winterianus*	*Haemophysalis longicornis*	Mortality	[177]
(Java citronella)	*R. microplus*	Mortality, inhibits reproduction	[178]
	*R. microplus*	Mortality, inhibits reproduction	[160]
*Drimys brasiliensis*	*R. microplus*, *R. sanguineus*	Mortality	[90]
(Tasmanian pepper leaf)			
*Eucalyptus citriodora*	*A. cajennense*, *A. nitens*	Mortality	[176]
(lemon-scented gum)	*I. ricinus*	Repellency/deterrence	[179]
*Gynandropsis gynandra*	*Rhipicephalus appendiculatus*	Repellency	[180]
(Shona cabbage)			
*Hesperozygis ringens*	*R. microplus*	Inhibits reproduction	[181]
(espanta-pulga)			
*Hyptis suaveolens*	*A. cajennense*	Repellency	[74]
(horehound)			
*Illicium verum*	*D. nitens*	Mortality	[174]
(star anise)			
*Juniperus occidentalis*	*I. scapularis*	Mortality	[163]
(western juniper)			
*Juniperus virginiana*	*I. scapularis*	Mortality	[97]
(eastern red cedar)			
*Lippia gracilis*	*R. microplus*	Mortality	[182]
(alecrimda-chapada)			
*Lippia graveolens*	*R. microplus*	Mortality	[183]
(Mexican oregano)			
*Lippia sidoides*	*Dermacentor nitens*	Mortality, inhibits reproduction	[184]
(pepper rosmarin)	*R. microplus*		
*Lippia triplinervis*	*R. microplus*	Mortality, inhibits reproduction	[185]
(no common name)			
*Melaleuca alternifolia*	*I. ricinus*	Mortality	[186]
(narrow-leaved paperbark)			
*Mentha piperita*	*Amblyomma hebraeum*	Repellency	[187]
(peppermint)			
*Mentha spicata*	*A. hebraeum*	Repellency	[187]
(spearmint)	*I. ricinus*	Repellency	[188]
*Nepeta cataria*	*R. appendiculatus*	Repellency	[189]
(catnip)			
*Ocimum basilicum*	*I. ricinus*	Repellency	[188]
(basil)			
*Ocimum suave*	*R. appendiculatus*	Mortality	[190]
(clove basil)			
*Origanum bilgeri*	*R. turanicus*	Mortality	[191]
(no common name)			
*Origanum majorana*	*I. ricinus*	Repellency	[188]
(majoram)			
*Origanum minutiflorum*	*Rhipicephalus turanicus*	Mortality	[192]
(Spartan oregano)			
*Origanum onites*	*R. turanicus*	Mortality	[193]
(Greek oregano)			
*Origanum vulgare*	*A. americanum*	Repellency	[194]
(oregano)			
*Pimenta dioica*	*R. microplus*	Mortality	[172]
(allspice)			
*Piper mikanianum*	*R. microplus*	Mortality	[128]
(pariparoba)			
*Piper tuberculatum*	*R. microplus*	Mortality	[164]
(cordoncillo)			
*Rosmarinus officinalis*	*I. ricinus*	Repellency	[188]
(rosemary)	*I. scapularis*	Mortality	[195]
	*R. microplus*	Mortality	[172]
*Tetradenia riparia*	*R. microplus*	Mortality, inhibits reproduction	[196]
(ginger bush)			
*Thymus sipyleus*	*R. turanicus*	Mortality	[197]
(no common name)			
*Zanthoxylum limonella*	*R. microplus*	Mortality	[112]
(prickly ash)			
*Zataria multiflora*	*R. annulatus*	Mortality	[155]
(za’atar)			
*Zingber officinale*	*R. microplus*	Mortality	[164]
(Canton ginger)			

## Data Availability

Not applicable.
